# How Did COVID-19 Change Faculty Members’ Use of Technology?

**DOI:** 10.1177/21582440221149720

**Published:** 2023-01-18

**Authors:** Nurullah Aydın, Muhammed Fatih Sayır, Süleyman Aydeniz, Tacettin Şimşek

**Affiliations:** 1 Atatürk University, Erzurum, Türkiye; 2 Muş Alparslan University, Türkiye

**Keywords:** COVID-19, use of technology, faculty members, after the end of pandemic

## Abstract

COVID-19 has caused great changes in education. Routines, practices, and especially the technologies used in teaching have differentiated at all levels of education. The purpose of this study is to determine the technologies used by faculty members for instructional purposes before and during the COVID-19 pandemic and their perspectives on the use of these technologies after the end of pandemic. A close-ended questionnaire was used for gathering data in this survey research study. Participants are150 faculty members from different ages and fields. Findings indicate that, although faculty members experienced various issues (e.g., infrastructure problems, access to technology, and lack of experience in technology) while teaching through technological facilities during the pandemic, they are in favor of using tools such as WhatsApp, Google classroom, Zoom Meeting, Facebook, and e-mail after the end of pandemic due to the different facilities they provide.

## Introduction

In the 90s, many researchers made various claims and predictions regarding the use of technology in education. “Technology can transform learning and teaching across a variety of existing curriculum areas: it is a very general educational resource” (Crook, 1996, p. 29). “Technology will play an increasingly prominent role in classroom instruction in the coming decades” and “New educational technologies promise to change forever the way students learn and the teachers teach yet again” (Kent & McNergney, 1999, p. 4). “Computer technology has a great potential in education” (Norman, 1994, p. 195). “Technology is frequently seen as academe’s “magic bullet,” the enabler of reforms that will silence higher education’s critics by making the academy more accessible, more affordable, and more effective” (Van Dusen, 1998, p. 59). “Instructional technologies have the potential to help higher education faculty address increasing demands on their time and energy” (Spotts et al.,1997). Today, regarding the use of technology in education the predictions and claims have come to live and even reached the imaginations.

As the technology develops, the use of technology in higher education institutions becomes diverse and widespread. Since the use of technology can offer new ways in teaching, it contributes to the acquisition of required knowledge and skills (Sailer et al., 2021). The fact that university students use technology at high levels offers higher education institutions the opportunity to use technology both as a communication and instruction tool (Ensminger & Lewis, 2011). The technological developments and the opportunities offered by higher education institutions have made the use of technology an inevitable phenomenon for the faculty members (DiVall et al., 2013), and they consider technology crucial for instruction (Spotts et al., 1997). According to faculty members, the use of technology in teaching contributes to increasing teacher knowledge, ease of communication, effective teaching, and flexible teaching (Khan et al., 2016). One of the turning points of technology use in higher education is the emergence of the Internet. Since the advent of the Internet and especially the World Wide Web, increasing use of technology in higher education has been encouraged by all stakeholders of higher education, and the public (Xu & Meyer, 2007).

The thoughts of faculty members appear as an issue that needs to be addressed, while underlying the use of technology in higher education. Various research shows that faculty members think the use of technology makes learning interesting to students, enriches their teaching and, contributes to their pedagogical development (Baran, 2016). Besides, faculty members consider it to be fundamental for their works (Nsouli & Vlachopoulos, 2021). Faculty members are in favor of using technology in their lectures (Gulbahar, 2008) and they also believe that the instructional technologies considerably enhance their teaching effectiveness (Peluchette & Rust, 2005). Faculty members have the intent to integrate technology in their current and future courses, and they also have positive attitudes toward the use of technology in instruction (Sagnak & Baran, 2021). Besides, faculty members need training on the use of technology efficiently (Hall & Elliot, 2003; Jong, 2019; Polly et al., 2021; J. Wang et al, 2017).

COVID-19, which emerged at the end of 2019 and has affected the whole world since the first months of 2020, has caused many changes in our lives. For example, the use of masks has become an indispensable part of lives of people. Face-to-face meetings have been substituted by online meetings even if the participants are in the same building. People could not go to the cinema or the theater due to the closures; even their shopping habits have changed (Sheth, 2020). COVID-19 has also caused great changes in education. Routines, practices, and especially the technologies used in teaching have differentiated at all levels of education.

Today, many technologies such as in-class tools (projection, smart board, and sound systems), online communication environments (Adobe connect, Google classroom, and Zoom meeting), and communication tools/social media environments (Facebook, WhatsApp, e-mail, and sms) are used in education. Faculty members prefer to use one or more than one of these technologies for instructional purposes. This study aimed to determine the technologies used by faculty members for instructional purposes before and during the COVID-19 pandemic and their perspectives on the use of these technologies after the end of pandemic. In addition, another goal of this study was to determine the problems related to use of technology experienced by faculty members during pandemic, the difficulties of using technology, and its conveniences. While analyzing the data, age variable was considered.

The current study is significant, because that it focuses on *after the end of pandemic* which refers to the return of faculty members and students to the pre-pandemic education environment in higher education. The literature argues that the studies conducted in this context have delved into the problems and solution proposals related to the use of technology during the pandemic process (Almazova et al., 2020; Bao, 2020; Garcia-Morales et al., 2021; Huang et al., 2020). However, this study has also examined the transfer of the experience gained during the pandemic process to the post-pandemic period. After the end of COVID-19, the change it caused in every field is a matter that needs to be examined. Based on this information the hypotheses of the present study are as follows:

1. The COVID-19 pandemic changes the educational technologies that faculty members used before the pandemic.
2. Faculty members experience problems regarding the technologies they use in their courses during the COVID-19 pandemic.
3. Technologies used by faculty members during the COVID-19 pandemic provide conveniences to them.
4. The technologies used in the courses during the COVID-19 pandemic cause problems.
5. Faculty members use the technologies, which they used in courses during the COVID-19 pandemic, after the end pandemic due to various reasons.

## Method

This is a survey research study. Survey research is a quantitative research in which researchers carry out a survey to a sample or to the entire population of people to describe the attitudes, opinions, behaviors, or characteristics of the population (Creswell, 2012). This study adopted a cross-sectional survey design, in which information is gathered at just a point of time, although the time it takes to collect all of the data may take anywhere from a day to a few weeks or more, from a sample that has been drawn from a predetermined population (Frankel et al., 2012).

## Participants

The participants were 150 faculty members working at different universities in Turkey, who were randomly selected with different ages and expertise in different fields. There were 35 people in the 25 to 35 age range, 56 people in the 35 to 45 age range, 34 people in the 45 to 55 age range, and 25 people in the 55 to 65 age range.

## Data Collection Instrument

A close-ended questionnaire, one of the most common used instruments for gathering data in survey research (Frankel et al., 2012), was applied to faculty members to reflect on their ideas about their use of technologies for instructional purposes before and during COVID-19 pandemic and their views on the use of these technologies after the end of pandemic. In the process of determining the questions in the questionnaire, first, the relevant literature was reviewed by the researchers, and a question pool consisting of fifteen questions was created. These questions were evaluated in terms of the purpose and content of the study by three faculty members who were experts in the field of measurement and evaluation. Based on expert suggestions, some questions were eliminated, and finally a seven-question questionnaire was created.

According to Frankel et al. (2012, p. 399) there are four practical standards that all survey questions should meet:

1. Is this a question that can be asked exactly the way it is written?
2. Is this a question that will mean the same thing to everyone?
3. Is this a question that people can answer?
4. Is this a question that people will be willing to answer, given the data collection procedures?

Two faculty members who were experts in the field of language examined the questions in the questionnaire according to these standards. The final form of the questionnaire was obtained by making some changes in the questions, taking into account the suggestions of these experts. The information of the questions listed in the questionnaire was as follows:

The first question in the questionnaire used as a data collection tool in the research was a single-choice question. It was aimed to determine how often the participants used technology in their courses before the pandemic with this question. The options of this question were *rarely*, *often*, and *always*.

The second question in the questionnaire was a multiple-choice question with three options. It aimed to determine the technologies used by faculty members in their courses before pandemic. The first option of this question was *in-class tools (projection, smart board, sound systems, etc.)*. The second option was *online communication environments (Adobe connect, Google classroom, Zoom meeting, etc.)*. The third option was *communication tools/social media environments (Facebook, WhatsApp, e-mail, sms etc.)*.

The third question was a multiple-choice question with three options. It aimed to determine the technologies used by faculty members in their courses during pandemic. The options for this question were the same as for the second question.

The fourth question was a multiple-choice question with three options. It aimed to determine the problems related to use of technology experienced by faculty members during pandemic. The first option of this question was *the problem of students’ access to technologies used in the courses*. The second option was *infrastructure problems (power outage, internet outage, internet quality, etc.)*. The third option was *lack of experience in using technology*.

The fifth question was a multiple-choice question with three options. It aimed to determine the conveniences provided by the use of technology to the faculty members during the pandemic. The first option of this question was *opportunity to communicate with students*. The second option was *directing students to information sources.* Third option was *removing the limitation in terms of time and space.*

The sixth question was a multiple-choice question with four options. It aimed to determine the problems that may arise in the courses conducted with technological opportunities during the pandemic. The first option of this question was *problems related to assessment and evaluation.* The second option was *communication problems caused by not being able to communicate face-to-face.* The third option was *ethical problems in online courses.* The fourth option is *the problem of lack of student-faculty member interaction during the course.*

The seventh question is an open ended question. It aimed to determine which of the technologies faculty members prefer to use after the end of pandemic that they used during pandemic. In this question, faculty members were also asked to explain the reasons of their preferences.

The close-ended questionnaire was formatted on a Google-form due to conditions caused by the COVID-19 pandemic and sent to faculty members via e-mail or WhatsApp. The questionnaire was started to be sent on 11.04.2021 and it was sent to 274 faculty members in total. 152 participants completed it and the data analysis started on 14.05.2021.

## Data Analysis

In the research, the data obtained from the questionnaire were analyzed by using the SPSS 25 statistical package program. In order to determine the opinions of the faculty members on the questions in the questionnaire, a descriptive analysis was performed by calculating the frequency and percentage for each item separately. In addition, in order to determine whether the opinions of the faculty members differ according to the age variable, difference analyzes were made using the chi-square test. However, in some cases, the Fisher-Freeman-Halton Exact test was used as an alternative, since the number of cells with the expected value less than 5 exceeded 20% of the total number of cells in the crosstabs. This test is the generalized version of the Fisher Exact test, which is used in cases where the assumptions of the Chi-square analysis are not met in 2 × 2 tables (Freeman & Halton, 1951; IBM, 2021). A significance level of .05 was taken into account in the interpretation of the results obtained from the statistical tests used in the study.

## Findings

Data analysis results for the questions in the questionnaire are as follows:

Table 1 presents the frequency of faculty members’ use of technology in their courses before the pandemic. According to statistical findings the frequency of faculty members’ use of technology in their courses before the pandemic does not show a statistically significant difference according to the age variable. However, when the frequency values and percentages in the table are taken into consideration, it is seen that the faculty members in the 55 to 65 age range use technology in their courses less than those in the other age groups before pandemic.

**Table 1.** The Frequency of Faculty Members’ Use of Technology in Their Courses Before the Pandemic.

Age	Rarely	Often	Always
25–35			
*f*	10	18	7
%	28.6	51.4	20.0
35–45			
*f*	16	31	9
%	28.6	55.4	16.1
45–55			
*f*	13	18	3
%	38.2	52.9	8.8
55–65			
*f*	11	10	4
%	44.0	40.0	16.0
Chi square test	χ^2^ = 4.00; *p* = .686		

The findings regarding to first hypothesis: The COVID-19 pandemic changes the educational technologies that faculty members used before the pandemic.

Table 2 presents technologies that faculty members used in their courses before pandemic. Participants’ answers to the options of *in-class tools* (χ^2^ = 1.98; *p* > .05) and *online communication environments* (χ^2^ = 1.32; p = *p* > .05) regarding the technologies they use in their courses before pandemic do not show a statistically significant difference according to the age variable. On the other hand, it is understood from frequency values and percentages in Table 2 that the faculty members prefer to use in-class tools in their classes, but they do not prefer to use online communication environments before pandemic. However, when the answers for the option of *communication tools/social media environments* is considered, a statistically significant difference was obtained according to the age variable (χ^2^ = 8.21; *p* < .05). Statistical findings show that as the age of faculty members increases, they use communication tools and social media environments less for educational purposes.

**Table 2.** Technologies That Faculty Members Used in Their Courses Before the Pandemic.

Age	In-class tools		Online communicatin environments		Communication tools/social media environments	
	Yes	No	Yes	No	Yes	No
25–35						
*f*	32	3	6	29	18	17
%	91.4	8.6	17.1	82.9	51.4	48.6
35–45						
*f*	52	4	7	49	21	35
%	92.8	7.2	12.5	87.5	37.5	62.5
45–55						
*f*	33	1	4	30	11	23
%	97.1	2.9	11.7	88.3	32.3	67.7
55–65						
*f*	22	3	5	20	4	21
%	88	12	20	80	16	84
Chi square test	χ^2^ = 1.98^a^; *p* = .592		χ^2^ = 1.32^a^; *p* = .746		χ^2^ = 8.21; *p* = .042*	

a Fisher-Freeman-Halton exact test.

* *p* < .05

Table 3 presents technologies that faculty members used in their courses during the pandemic. Participants’ answers to the options of *in-class tools* (χ^2^ = 1.67; *p* = .655) and *communication tools/social media environments* (χ^2^ = 1.29; *p* = .750) regarding the technologies they use in their courses during pandemic do not show a statistically significant difference according to the age variable. However, participants’ answers to the option of *online communication environments* show a statistically significant difference according to the age variable. The statistical findings regarding this option show that faculty members between the ages of 25 to 35 used online communication environments at a lower rate than those in other age groups. When the findings in Table 3 are evaluated in terms of frequency values and percentages, it is understood that faculty members used *online communication environments* and *communication tools/social media environments* in their classes during the pandemic.

**Table 3.** Technologies That Faculty Members Used in Their Courses During the Pandemic.

Age	In-class tools		Online communicatin environments		Communication tools/social media environments	
	Yes	No	Yes	No	Yes	No
25–35						
*f*	7	28	30	5	24	11
%	20	80	85.7	14.3	68.5	31.5
35–45						
*f*	17	39	56	0	33	23
%	30.3	69.7	100	0.0	59	41
45–55						
*f*	9	25	31	3	23	11
%	26.4	73.6	91	9.0	67.6	32.4
55–65						
*f*	5	20	24	1	14	11
%	20	80	96	4.0	56	44
Chi square test	χ^2^ = 1.67; *p* = .655		χ^2^ = 8.68^a^; *p* = .014*		χ^2^ = 1.29; *p* = .750	

a Fisher-Freeman-Halton exact test.

* *p* < .05.

When the findings in Tables 2 and 3 regarding the technologies used by faculty members in their classes before and during the pandemic are compared, it is seen that there are great changes. The findings show that during the pandemic *online communication environments* and *communication tools/social media environments* took the place of *in-class tools* which were widely used before the pandemic. One of the remarkable points is that the use of *online communication environments* and *communication tools/social media environments* mostly preferred by faculty members between the ages of 25 to 35 before the pandemic increased among other age groups during the pandemic.

The findings regarding to second hypothesis: Faculty members experience problems regarding the technologies they use in their courses during the COVID-19 pandemic.

Table 4 presents the problems related the use of technology experienced by faculty members during pandemic. Statistical data show that most of faculty members encountered *the problem of students’ access to technologies used in courses* (χ^2^ = 1.52; *p* = .697) and *infrastructure problems* (χ^2^ = 6.24; *p* = .103) also these findings do not show a statistically significant difference according to the age variable. However, the findings for the option of *lack of experience in using technology* (χ^2^ = 21.58; *p* = .000*) reveal that there is a statistically significant difference between the faculty members in the 45 to 55 and 55 to 65 age ranges and the faculty members in 25 to 35 and 35 to 45 age ranges in terms of lack of experience in using technology. Statistical findings show that faculty members between the ages of 45 to 55 and 55 to 65 suffer from lack of experience in using technology.

**Table 4.** The Problems Related to Use of Technology Experienced by Faculty Members During Pandemic.

Age	The problem of students’ access to technologies		Infrastructure problems		Lack of experience in using technology	
	Yes	No	Yes	No	Yes	No
25–35						
*f*	23	12	17	18	12	23
%	65.7	34.3	48.5	51.5	34.3	65.7
35–45						
*f*	40	16	38	18	20	36
%	71.4	28.6	67.8	32.2	35.7	64.3
45–55						
*f*	26	8	26	8	27	7
%	76.4	23.6	76.4	23.6	79.4	20.6
55–65						
*f*	16	9	18	7	17	8
%	64	36	72	28	68	32
Chi square test	*χ*^2^ = 1.52; *p* = .697		*χ*^2^ = 6.24; *p* = 0.103		*χ*^2^ = 21.58^a^; *p* = .000*	

a Fisher-Freeman-Halton exact test.

* *p* < .05.

Findings considering the third hypothesis: Technologies used by faculty members during the COVID-19 pandemic provide conveniences to them.

Table 5 presents conveniences provided by the use of technology to the faculty members during the pandemic. Statistical data reveal that the answers given to each option do not show a significant difference in terms of the age variable. When the data in Table 5 evaluated in terms of frequency values and percentages it is seen that, more than half of the faculty members in all age groups think that the use of technology during the pandemic period did not provide the opportunity to communicate with students. The frequency values and percentages also show that, more than half of faculty members in 25 to 35 and 35 to 45 age groups think the use of technology during pandemic facilitated directing students to information sources. On the other hand, the data for the option of removing the limitation in terms of time and space reveals that almost all of the faculty members in all age groups think that the use of technology during the pandemic removed the limitations in terms of time and space.

**Table 5.** Conveniences Provided by the Use of Technology to the Faculty Members During the Pandemic.

Age	Opportunity to communicate with students		Directing students to information sources		Removing the limitation in terms of time and space	
	Yes	No	Yes	No	Yes	No
25–35						
*f*	13	22	20	15	31	4
%	37.1	62.8	57.1	42.8	88.5	11.4
35–45						
*f*	26	30	33	23	51	5
%	46.4	53.5	58.9	41.1	91	9
45–55						
*f*	8	26	14	20	32	2
%	23.5	76.4	41.1	58.8	94.1	5.8
55–65						
*f*	10	15	12	13	24	1
%	40	60	48	52	96	4
Chi square test	χ^2^ = 3.96; *p* = .272		χ^2^ = 2.48; *p* = .481		χ^2^ = 1.36^a^; *p* = .714	

a Fisher-Freeman-Halton exact test.

* *p* < .05.

Findings associated with the fourth hypothesis: The technologies used in the courses during the COVID-19 pandemic cause problems.

Table 6 presents the problems that may arise in the courses conducted with technological opportunities during the pandemic. Statistical data reveal that the answers given to each option do not show a significant difference in terms of the age variable. When the data in Table 6 examined, it is seen that the faculty members experienced assessment and evaluation problems, communication problems, and the problem of lack of student-faculty member interaction in the courses they conducted with technological facilities during the pandemic. On the other hand, the findings regarding the option of ethical problems show that faculty members do not encounter ethical problems in online courses to a large extent.

**Table 6.** The Problems That May Arise in the Courses Conducted With Technological Opportunities During the Pandemic.

Age	Assessment and evaluation problems		Communication problems		Ethical problems		Lack of student-faculty ember interaction	
	Yes	No	Yes	No	Yes	No	Yes	No
25–35								
*f*	25	10	24	11	13	22	25	10
%	71.4	28.6	68.5	21.5	37.1	62.9	71.4	28.6
35–45								
*f*	42	14	48	8	25	31	48	8
%	75	25	85.7	14.3	44.6	55.4	85.7	14.3
45–55								
*f*	30	4	24	10	13	21	25	9
%	88.2	11.8	70.5	29.5	38.2	61.8	73.5	26.5
55–65								
*f*	18	7	20	5	11	14	20	5
%	72	28	80	20	44	56	80	20
Chi square test	χ^2^ = 2.47; *p* = .488		χ^2^ = 6.23; *p* = .104		χ^2^ = 1.07; *p* = .803		χ^2^ = 4.11^a^; *p* = .264	

a Fisher-Freeman-Halton exact test.

* *p* < .05.

The findings related to the fifth hypothesis: Faculty members use the technologies, which they used in courses during the COVID-19 pandemic, after the end pandemic due to various reasons.

The last question in the data collection tool was an open-ended question. It aimed to determine which of the technologies faculty members prefer to use after the end of pandemic that they used during pandemic with this question. In this question, faculty members were also asked to explain the reasons of their preferences. Content analysis method was used in the analysis of the data related to this question. During the content analysis process, five themes were created in line with the answers given by the faculty members to this question. These themes are ease of data and information exchange, opportunity of recording the course, the opportunity to invite guest faculty members to the course, providing convenience in terms of time and space, and the opportunity of easy communication with students.

### Ease of Data and Information Exchange

According to the data obtained on this theme, it is understood that the faculty members think that the technological opportunities such as Google classroom and WhatsApp that they use during the pandemic facilitate the exchange of data and information with students. Faculty members stated that they frequently used technological facilities such as Google classroom and WhatsApp during the pandemic period while giving homework, evaluating assignments and directing students to information sources, and stated that they intend to use these tools for these purposes after the end of pandemic. Some of the answers given by the faculty members that can be evaluated within the scope of this theme are as follows:

*I would like to use Classroom to share resources with students and to give homework and provide feedback with ease.*

*WhatsApp: It can be used for student assignment and information sharing.*

### The Opportunity of Recording Courses

When the data on this theme is examined, it is seen that the faculty members will continue to use tools such as Google classroom and Zoom meeting, as they provide the opportunity to record the courses. According to the faculty members, students who cannot attend a course for any reason will be able benefit from these records. The following answer can be evaluated within the scope of this theme:

*I will use Google classroom and zoom meeting as they offer students the opportunity to watch the lecture again.*

### The Opportunity to Invite Guest Faculty Members to the Course

Findings related to this theme show that faculty members invited other faculty members to their courses while using tools such as Google Classroom, Zoom Meeting, and Adobe Connect during the pandemic process. The faculty members’ answers such as *“I will use Zoom Meeting and Google Classroom as they provide the opportunity to host guest professors from other cities in the country and universities abroad.”* and “*With the Zoom program, we were able to bring other faculty members together with our students. Therefore, I think that this and similar programs should be used after the pandemic.”* reveal that the faculty members are considering to use these tools after the end of pandemic.

### Providing Convenience in Terms of Time and Space

When the faculty members’ answers examined, it was determined that most of them mentioned the speciality of technology providing convenience in terms of time and space. According to faculty members, tools such as Zoom Meeting and Google Classroom made great contributions to them by providing convenience in terms of time and space during the pandemic. It is understood from the answers such as *“I will use Zoom Meeting. Because it is very economical in terms of time and space.” “I plan to use applications such as Google Classroom, Zoom Meeting, Adobe Connect, which enable education to continue without time and place restrictions. The most important benefits of these are that they ensure continuity in education and communication with students. That’s why I find these tools very useful.”* and *“Zoom. It is an important advantage that it eliminates time and space limitations.”* that faculty members will use these tools after the end of pandemic as they provide convenience in terms of time and space.

### The Opportunity of Easy Communication With Students

One of the issues that faculty members most frequently mentioned while answering the seventh question is the opportunity of easy communication with students provided by technology. According to faculty members, communication tools such as WhatsApp, e-mail, and Facebook facilitated their communication with students during the pandemic, so they are considering using these tools after the end of pandemic. Faculty members’ answers such as *“The communication we established with the students via e-mail enabled them to gain a habit in this sense. I think this habit will continue.”* and *“I would like to continue to use social media tools such as Whatsapp to make communication fast and easy.”* show their intend to use these tools after the end of pandemic.

## Discussion

The results of the study reveal that the faculty members in the 55 to 65 age range use technology in their courses less than those in the other age groups before pandemic. Supporting this finding research argue that older faculty members use technology less than younger members (Xu & Meyer, 2007), and they exhibit less interest in using technology than their younger colleagues (Ahadiat, 2005). Older faculty members tend to show little or no conception change regarding technology use (Englund et al., 2017), so they are less likely to use technology while teaching (Meyer & Xu, 2009).

The findings indicate that the faculty members preferred to use in-class tools compared to online communication environments, communication tools, and social media environments before the pandemic. However, while the use of in-class tools has decreased, there has been an increase in the use of online communication environments (e.g., Google Classroom and Zoom Meeting) during the pandemic. The literature indicates that due to the closures and limitations caused by the pandemic, faculty members tend to adopt these environments that can be used as distance education tools (Cahyadi et al., 2022; Singh et al., 2021; Turnbull et al., 2021). The striking finding is that an increase was observed in the use of communication tools and social media environments that were less used by older faculty members before the pandemic.

Another finding is that most of faculty members encountered infrastructure problems and the problem of students’ access to the technology used in courses. This confirms the literature. The quality of internet connection and interruptions, which can be evaluated within the scope of infrastructure problems, are the most common problems experienced by faculty members regarding the use of technology in their courses during the pandemic (Ahmed & Opoku, 2021; Khalil et al., 2020, Mishra et al., 2020). The first studies on pandemic, examined the issue of students’ access to technology in their courses, underlying the importance of this issue (Ali, 2020, Beaunoyer et al., 2020, Crawford et al., 2020). Similar to current study Adarkwah (2021) and Khatoony and Nezhadmehr (2020) have reported the problem related to students’ access to the technology in their courses during pandemic. On the other hand, the statistical findings of study reveal that faculty members between the ages of 45 to 55 and 55 to 65 suffered from of lack of experience in using technology. This shows that the problem of lack of experience in technology use among older faculty members continues during the pandemic period.

Based on the views of faculty members on the convenience of technology use during the pandemic period, almost all of them think that technology use removes limitations in terms of time and space. Various studies have focused on this advantage of technology (Al Kurdi et al., 2020; Bakhov et al., 2021, Huang et al., 2020; S. Wang et al., 2021). Xie et al. (2020) highlighted the flexibility provided in terms of time and space as the first convenience provided by the courses conducted with technological facilities during pandemic. The findings of the study display that faculty members also believe that technological opportunities provide great convenience in communicating with students and directing them to information sources.

The findings of the study indicate that faculty members had problems related to measurement and evaluation. Although assignments were the most used tools and students were generally satisfied about the quality of the assessment practices (Senel & Senel, 2021), faculty members had problems in the field of measurement and evaluation during the pandemic such as increased time required to complete the exam setup (Khalaf et al., 2020), academic dishonesty, infrastructure, coverage of learning outcomes, and commitment of students to submit assessments (Guangul et al., 2020), and lack of technical infrastructure, limited awareness of online teaching platforms and security concerns (Joshi et al., 2020).

One of the issues that the current study focuses on is to determine which of the technologies faculty members plan to use after the end of pandemic that they used during the pandemic. Faculty members were also asked to indicate the reasons for their preferences. Faculty members consider using;

1. WhatsApp and Google Classroom applications after the pandemic as they provide the ease of data and information exchange.
2. Google Classroom and Zoom Meeting as they provide the opportunity of recording courses.
3. Google Classroom, Zoom Meeting, and Adobe Connect as they provide the opportunity to invite guest faculty members to the course.
4. Zoom Meeting and Google Classroom as they provide convenience in terms of time and space.
5. WhatsApp, e-mail, and Facebook as they provide the opportunity of easy communication with students.

## Conclusion

The pandemic has changed the technological tools that faculty members use in their courses. During the pandemic, problems (e.g., infrastructure problems, access to technology, and lack of experience in technology use) were encountered in the courses conducted with technological facilities. Despite these negativities, many faculty members started to benefit from technologies that they used very little before and realized the opportunities they provide. The findings of the present study reveal that the faculty members plan to use tools such as WhatsApp, Google Classroom, Zoom Meeting, Facebook, and e-mail after the end of pandemic due to the different facilities they provide. As a result, it is understood that the pandemic may cause changes in the technologies that faculty members use in their courses.
